# PW02-019 - Inflammatory pathways activation in TRAPS patients

**DOI:** 10.1186/1546-0096-11-S1-A159

**Published:** 2013-11-08

**Authors:** O Negm, EM McDermott, E Drewe, R Powell, F Lucy, T Ian, P Tighe

**Affiliations:** 1 Immunology, School of Molecular Medical Sciences, University of Nottingham, UK; 2 Nottingham University Hospitals, National Health Service Trust, Nottingham, UK

## Introduction

Mutations in TNFRSF1A can result in the autosomal dominant TNF receptor-associated periodic syndrome

(TRAPS): a complex and heterogeneous systemic autoinflammatory disorder. Misfolding, intracellular aggregation and ligand-independent signalling by mutant TNFR1 play central roles in disease pathophysiology.

## Objectives

This work was conducted to study the intracellular signalling pathway activation elicited by mutant TNFR1.

## Methods

To understand the complexity of intracellular signalling pathway perturbation in TRAPS, a prototypic mutant TNFR1 (C33Y), or wild-type TNFR1 (WT), were expressed at near physiological levels in an SK-Hep-1 cell model system. TNFR1-associated signalling pathway intermediates were examined under a range of conditions, employing reverse-phase protein microarray. Peripheral blood mononuclear cells (PBMC) from C33Y TRAPS patients and matched healthy controls were similarly examined.

## Results

In comparison to cells expressing WT TNFR1 alone, expression of C33Y-TNFR1 in SK-Hep–1 cells and TRAPS patients’ PBMCs revealed a subtle up-regulation of a wide spectrum of signalling intermediates and their phosphorylated forms. These were associated with a proinflammatory/ anti-apoptotic phenotype, including NF-**k** B, p38, MEK/ERK and JNK MAP kinase pathways, Phosphoinositide 3 kinase, STAT3, JAK2/c-Src, Gsk-3**β** and transcription factors (including ATF, Elk, Jun). Increased activated Jak2/STAT3 may contribute to an “IL6 amplifier” positive feedback loop that promotes and sustains a proinflammatory state.

## Conclusion

The study thus reveals the pleiotropic effect of a TRAPS-associated mutant form of TNFR1 on multiple inflammatory signalling pathways.

## Disclosure of interest

None declared.

